# Management of sleep-disordered breathing in achondroplasia: guiding principles of the European Achondroplasia Forum

**DOI:** 10.1186/s13023-025-03717-0

**Published:** 2025-05-15

**Authors:** Brigitte Fauroux, Moeenaldeen AlSayed, Tawfeg Ben-Omran, Silvio Boero, Mieke Boon, Valérie Cormier-Daire, Svein Fredwall, Encarna Guillen-Navarro, Melita Irving, Philip Kunkel, Núria Madureira, Mohamad Maghnie, Josef Milerad, Klaus Mohnike, Geert Mortier, Lino Nobili, Zagorka Pejin, Marco Sessa, Sérgio B. Sousa

**Affiliations:** 1 AP-HP Necker-Enfants Malades University Hospital and Paris Cité University, and EA 7330 VIFASOM, Paris University, Paris, France; 2 King Faisal Specialist Hospital and Research Centre in Riyadh, Riyadh, Kingdom of Saudi Arabia; 3 Division of Genetics and Genomic Medicine, Sidra Medicine & Hamad Medical Corporation, Doha, Qatar; 4 Istituto Giannina Gaslini, Genoa, Italy; 5 Department of Pediatrics, University Hospital Leuven, Leuven, Belgium; 6 TRS National Resource Centre for Rare Disorders, Sunnaas Rehabilitation Hospital, Nesodden, Norway; 7 Medical Genetics Division & Pediatrics Department, Virgen de la Arrixaca University Hospital, IMIB-Pascual Parrilla, University of Murcia, CIBERER-ISCIII, Murcia, Madrid, Spain; 8 Guy’s and St Thomas’ NHS Foundation Trust, London, UK; 9 University Medical Centre Mannheim, Mannheim, Germany; 10 Hospital Pediátrico de Coímbra, Unidade Local de Saúde de Coímbra, Coimbra, Portugal; 11 IRCCS Istituto Department of Neuroscience, Rehabilitation, Ophthalmology, Genetics, Maternal and Child Health, University of Genoa, and Pediatric Endocrinology Unit, IRCCS Giannina Gaslini, Genoa, Italy; 12 Paediatric Sleep Research Unit, Astrid Lindgren´S Children’s Hospital, Östgötagatan 100, Box 4700, 116 92 Stockholm, Sweden; 13 Children’s Hospital, Otto-Von-Guericke-University, Magdeburg, Germany; 14 Center for Human Genetics, University Hospitals Leuven and KU Leuven, Leuven, Belgium; 15 IRCCS Istituto G. Gaslini, DiNOGMI, University of Genoa, Genoa, Italy; 16 Italian Association on Achondroplasia, Bari, Italy

**Keywords:** Achondroplasia, European Achondroplasia Forum, Obstructive sleep apnoea, Central sleep apnoea, Alveolar hypoventilation, Consensus, Sleep-disordered breathing

## Abstract

Due to the craniofacial anatomy of people with achondroplasia, sleep-disordered breathing (SDB) occurs more frequently than in the average stature population. SDB, which comprises obstructive sleep apnoea (OSA), more rarely central sleep apnoea (CSA), and nocturnal alveolar hypoventilation (NH), may present at any age in patients with achondroplasia. Untreated SDB is associated with neurocognitive dysfunction, cardiovascular, and metabolic complications in children and adults. There continues to be debate on the optimal assessment and management of SDB in achondroplasia. To help address this, the European Achondroplasia Forum (EAF), a network of clinicians and patient advocates representative of the achondroplasia clinical community, organised a virtual workshop in October 2023 to scrutinise, vote and agree upon five guiding principles for managing SDB in achondroplasia. This workshop was attended by 40 healthcare professionals, including clinical geneticists, general practitioners and consultants, orthodontic and orthopaedic surgeons, paediatricians, paediatric endocrinologists and pulmonologists, sleep researchers and specialists, and two patient advocacy group representatives. The five guiding principles focus on lifelong assessment and proactive management, incorporating individualised sleep studies, screening, and a stepwise approach to therapeutic management. The EAF was in favour of all guiding principles, with all achieving 100% consensus with high levels of agreement (range 8.9–9.7/10). In developing guiding principles for the management of SDB in achondroplasia, the EAF aims to facilitate optimal screening and management of SDB in infants, young children, and adults with achondroplasia.

## Background

Achondroplasia is the most common form of skeletal dysplasia, primarily characterised by disproportionate short stature and craniofacial malformation [1, 2]. Caused by a variant in the fibroblast growth factor receptor 3 (*FGFR3*) gene, the worldwide birth prevalence of achondroplasia is estimated to be approximately 3.72–4.6 per 100,000 [2–4]. Achondroplasia is a complex condition with multisystem involvement and a risk of musculoskeletal, neurological, cardiorespiratory, and ear, nose, and throat (ENT) complications which may occur across the lifespan [1, 2, 5].

Sleep-disordered breathing (SDB) encompasses obstructive sleep apnoea (OSA) and other less frequent sleep disorders such as central sleep apnoea (CSA), and nocturnal alveolar hypoventilation (NH) [6–11]. The prevalence of OSA in the general adult population ranges from 4 to 6%, whereas the frequency of OSA in adults with achondroplasia may be as high as 60% [12–14]. While SDB in achondroplasia has been extensively studied, data on other skeletal dysplasias are more limited. However, a literature review showed that SDB may be similarly prevalent across other skeletal dyplasias, including osteogenesis imperfecta, craniosynostosis, and Ellis-van Creveld syndrome [15, 16]. Patients with achondroplasia are at high risk of OSA because of anatomical abnormalities of the craniofacial bones with mid-face hypoplasia and nasopharyngeal airway narrowing [17]. Infants with achondroplasia are particularly at risk of OSA, and CSA because of brain stem dysfunction due to cervico-occipital compression [18]. There is also an increased incidence of sudden unexpected deaths in childhood in this patient population [2, 7, 18–23]. Older children and adults remain at risk of OSA as craniofacial abnormalities persist and may worsen with age [17]. SDB may be aggravated, and accompanied by NH, due to the relative chest hypoplasia and progressive scoliosis, as well as increased weight or obesity, which is common in patients with achondroplasia [24–26]. OSA is associated with neurocognitive dysfunction, abnormal behaviour, metabolic syndrome, and cardiovascular stress [27–29]. The early diagnosis and treatment of SDB is thus of paramount importance. Unfortunately, clinical symptomatology, examination findings, or sleep questionnaires are not sufficiently reliable on their own to diagnose OSA, CSA and NH. As such, there is a need for systematic screening with sleep studies, which should be accessible for all patients with achondroplasia. Patient management and follow up should be performed by a multidisciplinary achondroplasia team in order to diagnose and treat SDB in a timely manner, thereby avoiding the deleterious consequences and sequelae associated with persistent SDB.

## The European Achondroplasia Forum

The European Achondroplasia Forum (EAF) is a network of specialists in achondroplasia management. Working in collaboration with healthcare professionals and representatives of patient advocacy groups, the aim of the EAF is to improve overall care for individuals with achondroplasia through cross-country sharing of best practices and development of recommendations. The EAF is led by a Steering Committee comprised of clinical geneticists, paediatric endocrinologists, orthopaedic surgeons, paediatricians, an adult care specialist, a paediatric neurologist a sleep specialist, a paediatric pulmonologist, and a neurosurgeon, and a patient advocacy group representative, with a representation from 10 countries across Europe and the Middle East [30].

### Aims of developing guiding principles for the management of SDB

Early recognition and proper treatment of SDB are crucial in achondroplasia to avoid neurocognitive, cardiovascular, and metabolic complications [7]. Yet, despite these recognised lifelong consequences and concerns, there continues to be debate on the optimal assessment and management of SDB [24]. Untreated SDB may have serious developmental consequences in children with achondroplasia [1]. It is therefore essential that SDB is detected early and correctly managed. To address this and the lack of guidance in the literature, the EAF aimed to achieve consensus on screening and managing SBD in individuals with achondroplasia, providing guiding principles for optimal care.

### Developing guiding principles for the management of SDB

Following a search of the literature for recommendations on assessing and managing SDB in achondroplasia, five guiding principles were developed for discussion at an EAF open workshop held in October 2023. Attendees were invited to participate in the virtual workshop via the EAF website, mailing list and promotion through the Steering Committee and their network of contacts. The workshop was attended by representatives from representatives from 17 countries, and included 40 healthcare professionals (clinical geneticists, general practitioners and consultants, orthodontic and orthopaedic surgeons, paediatricians, paediatric endocrinologists and pulmonologists, sleep researchers and specialists) and two patient advocacy group.

Workshop participants discussed and scrutinised the draft guiding principles, alongside current practices, considering content, wording, support from the literature and collective expert opinion. Anonymous live voting was held to establish consensus and the level of agreement for each principle. The voting process adhered to the EAF Standard Operating Procedure for Developing Guiding Principles/Recommendations [26], in which a majority of 75% in favour is needed to pass the principle at the first vote. Once a principle receives ≥75% consensus, a level of agreement is established on a scale of 1–10, where 1 is ‘do not agree at all’, and 10 is ‘strongly agree’.

Five guiding principles were agreed upon [Table 1]. The first covers the need for lifelong assessment and proactive management of SDB in achondroplasia (A); the second emphasises that assessment for SDB should be tailored to the needs, age and symptoms of the individual (B); this is followed by guiding principles that focus on routine screening for SDB throughout life (C); undertaking and interpreting sleep studies and therapeutic management within the multidisciplinary team (MDT) (D); and individualising therapeutic management based on the age of the patient and the type and severity of SDB (E). After scrutiny and some amendments to the wording of one principle (principle D), all five guiding principles achieved 100% consensus, with high levels of agreement (range 8.9–9.7).

**Table 1:** Sleep-disordered breathing guiding principles

Item	Guiding principle	Vote (%)	Level of agreement (mean; range)
A	Due to the high prevalence of sleep-disordered breathing in individuals with achondroplasia of all ages, and the risk of associated complications, lifelong assessment and proactive management is required	100%	8.9 (5–10)
B	The type of sleep study required to assess for SDB should be individualised to the age, needs, and symptoms of the patient	100%	8.9 (6–10)
C	Screening for SDB should be undertaken routinely throughout the life course, with particular focus on the early years of life when onset is more common	100%	9.1 (7–10)
D	If SDB is suspected, a sleep study should be performed, with scoring made by a sleep specialist as soon as possible; interpretation and therapeutic management should be performed within an achondroplasia MDT	100%	9.5 (7–10)
E	Stepwise therapeutic management should be individualised to the type and severity of SDB and the age of the patient	100%	9.7 (8–10)

**A. Due to the high prevalence of sleep-disordered breathing in individuals with achondroplasia of all ages, and the risk of associated complications, lifelong assessment and proactive management is required.**

Summary of Key Points:

- The abnormal anatomy of the face and upper airways, along with increased rates of increased weight in achondroplasia underpin the high frequency of OSA.
- OSA is often more frequent and more severe in patients with achondroplasia than in the general population.
- Prevalence of CSA is relatively low in infants with achondroplasia.
- Patients with achondroplasia are likely to be more at risk of NH than the general population.
- Due to the serious consequences of OSA in infants and children with achondroplasia, close monitoring and systematic sleep studies are required.
- As untreated OSA can cause metabolic syndrome and cardiovascular stress, lifelong assessment and proactive monitoring are vital in patients with achondroplasia, particularly in adult patients.

Patients with achondroplasia may have mid-face hypoplasia, maxillary retrusion and associated dental malocclusion [17]. A recent study investigating 2D cephalometrics and 3D geometric morphometry analyses in 15 children with achondroplasia showed constant maxillo-mandibular retrusion with excessive vertical dimensions of the lower third of the face and modifications of cranial base angles. Patients may also have decreased temporomandibular joint mobility and upper airway muscle hypotonia [10]. These anatomical particularities reduce the calibre of the upper airway, especially during sleep; an anatomo-functional study revealed a significant positive correlation between maxillo-mandibular retrusion and OSA severity (*p* < 0.01) [17]. Infants and young children are particularly at risk of OSA because of their small upper airways. In this age group, OSA may be precipitated by viral infections of the upper airways which promote hypertrophy of the adenoids and tonsils, further impairing the upper airway. The risk of OSA persists throughout life; 3D morphometric analyses showed an aggravation of the craniofacial phenotype with age, predominantly affecting the midface, with increasing maxillary retrusion [17]. Increased weight or obesity, which are known risk factors for OSA in healthy individuals, are frequent in children and adults with achondroplasia [12].

The literature demonstrates a prevalence ofOSA in 56–60% of infants and children with achondroplasia, compared to 2–6% in the general population [12–14, 31, 32].

OSA is usually more severe in children and adults with achondroplasia, with a significantly higher apnoea–hypopnoea index (AHI), compared to subjects without an underlying condition. Mild, moderate, and severe OSA still occur in those children who had previously received upper airway surgery, including adenotonsillectomy, adenoturbinectomy, or both [10]. This is also the case in adults [12], highlighting that previous upper airway surgery does not preclude OSA in individuals with achondroplasia.

Achondroplasia is characterised by skull and spine abnormalities with cervical cord compression being common during the first months of life [10, 11, 20, 33]. Occipito-cervical compression, due to foramen magnum stenosis, may cause brain stem dysfunction, myelomalacia, and CSA. However, the prevalence of CSA is relatively low in infants with achondroplasia, showing that the brain stem, at least the central respiratory drive, remains preserved in infants and children, even in those with occipitocervical decompression. No correlation has been observed between the foramen magnum dimension or any other radiological parameter on magnetic resonance imaging (MRI), clinical symptoms or the central apnoea index (CAI) on polysomnography (PSG), underlining the need of systematic PSG/cardio-respiratory polygraphy (PG) [34].

Patients with achondroplasia are at risk of restrictive lung disease and therefore NH due to chest hypoplasia, respiratory muscle hypotonia and chest and spinal deformity. NH may co-exist or be present independently of OSA or CSA. Indeed, because of the relatively small thorax, patients adopt a rapid shallow breathing, i.e. rapid breathing with a small tidal volume, which may cause insufficient removal of carbon dioxide (CO_2_) and subsequent NH. For a similar AHI or CAI, the risk of concomitant NH is thus greater for patients with achondroplasia than for healthy controls. As the monitoring of CO_2_ is not performed routinely in every sleep laboratory, the real prevalence of NH is likely underestimated. However, the more severe the SDB, the higher the risk of NH [10].

Undiagnosed OSA and CSA during infancy can increase the risk of sudden death, which occurs in 2–5% of infants with achondroplasia [35, 36]. Therefore, close monitoring with systematic sleep studies during the first 2 years of life is crucial, in order to diagnose SDB in a timely manner [23, 36, 37]. Later in childhood, as in healthy children, undiagnosed and untreated OSA may cause poor academic performance with concentration difficulties, abnormal behaviour with irritability, daytime fatigue, enuresis, and morning headache [27–29]. These deleterious consequences of OSA have been extensively reported in healthy children but data in children with achondroplasia are scarce. In a study of 30 consecutive children with achondroplasia, impaired concentration at school was observed in four of eight children older than 6 years [9]. Due to concomitant factors such as hearing loss, which may contribute to neurocognitive dysfunction in children with achondroplasia, the real effect of OSA and the benefit of its treatment is difficult to ascertain.

Untreated OSA may cause metabolic syndrome and cardiovascular stress in children, but to a lesser extent than in adults. A Norwegian population study [12] found that the prevalence of hypertension was significantly higher in adults with achondroplasia and OSA (48%) compared to those without OSA (15%). With documented evidence of association between OSA and increased risk of cardiovascular disease [38], lifelong assessment and proactive management are vital. Consequently, all healthcare professionals and parents should be informed about the high prevalence and symptoms of SDB [35].

## B. The type of sleep study required to assess for sleep-disordered breathing should be individualised to the age, needs, and symptoms of the patient.

Summary of Key Points:

- Overnight, attended, in-laboratory PSG represents the gold standard for the diagnosis of and characterisation of SDB.
- When PSG is not available, alternatives such as ambulatory PSG, PG, or at minimum SpO_2_ with transcutaneous carbon dioxide pressure (PtcCO_2)_ may be suggested.
- In order to ensure quality of signal is maintained during a sleep study, studies should be performed by a sleep laboratory team.
- Equipment used should be validated and adapted for the patient’s age and needs.

An overnight, attended, in-laboratory PSG represents the gold standard for the diagnosis of any type of SDB. PSG gives information on the type and number of respiratory events, but also on sleep duration, sleep architecture with sleep stages, and sleep quality, and should be utilised in infants and young children. However, PSG is expensive, its access is limited, and the reading is time-consuming. For this reason, when PSG is not available or not feasible other types of sleep studies may be an alternative, such as PG, which records only the respiratory signals without the electroencephalographic (EEG) signals [22, 23]. A PG is usually better accepted by the patient but has the limits that it may underscore the hypopnea index because it is not possible to score hypopneas that are not accompanied by a cortical arousal. Ambulatory PSG or PG may be proposed by some centres in selected and cooperative patients. Overnight recording of pulse oximetry (SpO_2_) and PtcCO_2_ constitutes the simplest alternative to a PSG/PG, but these signals are limited by a low sensitivity and only give information on the consequences of SDB on nocturnal gas exchange with no information on the type, number, and severity of respiratory events.

The quality of the signal is of great importance to have a sufficient period with scorable signals [39]. Infants with achondroplasia are usually tachypnoeic; hypoxemia and hypercapnia are more common in these children compared to older patients and healthy counterparts.

It is important to use equipment that is validated for the patient’s age and adapted to the patient’s constitution; not all devices can be used in children less than 2 years of age [39]. To avoid the underdiagnosis of NH, systematic overnight recording of PtcCO_2_ is recommended [9].

It is therefore recommended for sleep studies to be performed by a sleep laboratory team with expertise in paediatric sleep; scoring should be performed by a specialist expert in paediatric sleep and, ideally, also in achondroplasia.

## C. Screening for sleep-disordered breathing should be undertaken routinely throughout the life course, with particular focus on the early years of life when onset is more common.

Summary of Key Points:

- Nocturnal and diurnal symptoms, sleep questionnaires, and clinical upper airway examination cannot be relied upon for diagnosis of SDB; a sleep study is required for accurate diagnosis of SDB.
- The EAF proposes screening for OSA routinely throughout life, with a focus on early years. Screening should be performed by an achondroplasia MDT, working with a local healthcare team. There are currently no recommendations for assessing SDB in adults with achondroplasia.

OSA may be associated with a wide range of nocturnal and diurnal symptoms in healthy children as well as those with achondroplasia. The most common clinical symptoms during sleep are frequent or permanent snoring, witnessed apnoea, mouth breathing, hyperextension of the neck, night sweats, nocturnal awakenings, and restless sleep [9, 22, 35]. Daytime symptoms comprise a decrease in academic performance, poor attention, including new onset of distractibility, irritability and abnormal behaviour, daytime fatigue, and sleepiness in older children [1]. Growth failure or hypertension may be a clinical manifestation in young children with OSA [23]. However, these symptoms are not specific and are insufficiently reliable for the diagnosis of OSA. Sleep questionnaires lack specificity and sensitivity for the diagnosis of OSA in patients with a craniofacial anomaly such as achondroplasia, even if they are combined with clinical symptoms and/or imaging [9, 40–42]. Clinical examination of the upper airways is neither able to predict or diagnose OSA [40–42]. Consequently, the diagnosis of OSA can only be made on an overnight PSG or PG [9]. Sleep questionnaires may help prioritise patients for sleep study [1], and a history of upper airway surgery does not preclude the presence of OSA [9].

With prevalence estimates for OSA in both children and adults with achondroplasia as high as 60% [12, 13, 17, 30], the EAF proposes screening for OSA in patients with achondroplasia routinely throughout life. Although OSA may present at any age, there should be a particular focus on the early years when OSA is more common [1, 23, 35]. The achondroplasia MDT, working in parallel with the local healthcare team, should undertake regular evaluation of infants in the first year of life [35], with continued close monitoring during the first 2 years [23, 35, 37].

If symptoms are present in children aged 1 to 18 years with an underlying disease associated with an increased risk of OSA (such as achondroplasia), the European Respiratory Society Task Force recommends screening via PSG [23]. International consensus recommends a PSG should be completed during the first year of life for all infants with achondroplasia [35].There are currently no specific recommendations for assessing OSA in adults [12]. The bed partner should be informed about the symptoms that suggest OSA. Compared to OSA, CSA is usually asymptomatic or pauci-symptomatic [43]. There are no questionnaires to screen for CSA. This is also the case for NH with moderate levels of NH being asymptomatic. It is only in cases of severe NH that the patient may complain of daytime headaches, fatigue, nausea, and dizziness [43].

**D. If sleep-disordered breathing is suspected, a sleep study should be performed, with scoring made by a sleep specialist as soon as possible; interpretation and therapeutic management should be performed within an achondroplasia MDT.**

Summary of Key Points:

- Sleep specialists assessing sleep study results from patients with achondroplasia do not need to be experts in achondroplasia, but an expert in achondroplasia should be available to review the results.
- Interpretation and therapeutic management of SDB should be performed within an achondroplasia MDT.
- Skeletal dysplasia MDTs should aim to include a sleep specialist.

This guiding principle was originally worded ‘If SDB is suspected, referral to a specialist should be made as soon as possible; sleep study results should be assessed by a specialist with knowledge of the respiratory characteristics of achondroplasia specific to the age of the patient’. Through discussion, the group agreed that a sleep specialist did not necessarily need to be also an expert in achondroplasia, but that an achondroplasia expert needed to be available to discuss the results of the sleep study. In line with previous EAF guiding principles [37] the importance of carrying out, interpreting, and discussing results as part of a wider achondroplasia MDT was deemed essential. It was also noted that the question asked of a sleep specialist was key to ensuring the sleep study and results are appropriate. Every skeletal dysplasia MDT should aim to integrate a sleep specialist who progressively develops experience in the variety of challenges that affect this population. As such, the statement was reworded prior to voting to ‘If SDB is suspected, a sleep study should be performed, with scoring made by a sleep specialist as soon as possible; interpretation and therapeutic management should be performed within an achondroplasia MDT’.

## E. Stepwise therapeutic management should be individualised to the type and severity of sleep-disordered breathing and the age of the patient.

Summary of Key Points:

- It is highly recommended to perform a sleep endoscopy before every surgical procedure, with overnight observation recommended for any child with achondroplasia and OSA undergoing upper airway surgery.
- Patients with achondroplasia and OSA should undergo a control sleep study 2 to 4 months after upper airway surgery.
- Should OSA persist in a patient with achondroplasia following upper airway surgery, orthodontic treatment, maxillofacial surgery, or continuous positive airway pressure (CPAP) should be discussed with the achondroplasia MDT team.
- While CSA can be resolved with cervico-occipital decompression; in case of NH, correction of OSA may resolve NH; if not, NIV is indicated.

There is international consensus that adenotonsillectomy should be first-line treatment for OSA in children with achondroplasia [35]. However, some children may have other obstructive sites, such as a hypertrophy of the turbinates or the lingual tonsils in older children. For this reason, it is highly recommended to perform a drug-induced sleep endoscopy before every surgical procedure to treat all the obstructive sites [43, 44]. Due to an increased risk of cardiorespiratory complications, overnight inpatient observation is recommended for any child with achondroplasia and OSA undergoing upper airway surgery [1, 45, 46]. It is mandatory to reevaluate OSA by a sleep study approximately 2 to 4 months after the upper airway surgery due to an increased risk of residual OSA after upper airway surgery [1, 35]. In a retrospective review of 95 children with achondroplasia, 18% (22/34) of those who underwent adenotonsillectomy as the initial procedure required further surgery [45].

In children with achondroplasia who have residual OSA following upper airway surgery, further treatment options include orthodontic and/or maxillofacial surgery with mid-face advancement, or non-invasive CPAP when all the above-mentioned therapies are not indicated, not wanted by the patient or caregivers, or are unsuccessful [1, 35]. CPAP has the advantage of being a non-invasive multi-level treatment of all obstructive sites during sleep. It may be performed at any age, from newborn to adult, thanks to the availability of devices and interfaces for every age. The main side effect of CPAP is that long-term use may favour maxillary retrusion, underlining the need for a systematic maxillofacial follow up and the regular evaluation of other, more definitive therapeutic options. Facial deformity is limited with nasal pillows or nasal canula, but these interfaces are not available for young children. Patients with increased weight or obesity may benefit from weight loss interventions [1, 47]. The role of upper airway surgery in adults with OSA remains unclear [1]. Tracheostomy is a final option for the treatment of severe persistent OSA. Fortunately, thanks to the management by a MDT, the indication for tracheostomy is exceptional.

Treatment for CSA involves cervico-occipital decompression. For NH, the first step is the optimal correction of OSA. If NH persists despite optimal correction of OSA, or if NH is present without OSA, non-invasive, bilevel ventilation (NIV) is indicated. As with CPAP, NIV should be initiated and followed by a paediatric expert in CPAP and NIV.

## Conclusions

SDB is rarely an acute life-threatening problem in patients with achondroplasia, however it may have considerable long-term deleterious consequences through the lifespan that can be mitigated [1]. This is especially relevant in infants and children with achondroplasia, when early recognition and management are crucial to avoid neurocognitive, cardiovascular, and metabolic complications [7]. By taking a proactive approach to managing OSA, the symptoms that affect daily life such as negative learning, behavioural consequences, daytime fatigue, disrupted growth hormone secretion and increased risks of accidents can be reduced, leading to an increased quality of life [1].

To date, there has been no specific guidance based solely on identifying, managing, and treating SDB in patients with achondroplasia. In developing guiding principles for the diagnosis and management of SDB, the EAF aims to provide healthcare professionals with overarching considerations that may be implemented in any healthcare system. It is hoped that by improving clarity on the impact of SDB in achondroplasia, infants, young children, and adults will receive optimal screening and management. Communicating and disseminating the guiding principles within the achondroplasia community will be an important step to improve overall patient care and quality of life, and assessing the impact of this guidance will be valuable. These guiding principles must be reviewed and updated in the future, according to developments in the field that may improve clinical practice, to ensure they remain relevant to the achondroplasia clinical community.

## Data Availability

All available data have been shared.

## Abbreviations

AHI: Apnoea–hypopnoea index
CAI: Central apnoea index
CSA: Central sleep apnoea
CPAP: Continuous positive airway pressure
EAF: European achondroplasia forum
EEG: Electroencephalographic
ENT: Ear, nose, and throat
*FGFR3*: Fibroblast growth factor receptor 3
MDT: Multidisciplinary team
NH: Nocturnal alveolar hypoventilation
NIV: Non-invasive ventilation
OSA: Obstructive sleep apnoea
PtcCO_2_: Transcutaneous carbon dioxide pressure
PG: Polygraphy
SDB: Sleep-disordered breathing
SpO_2_: Pulse oximetry
